# Market Intelligence and Incentive-Based Trait Ranking for Plant Breeding: A Sweetpotato Pilot in Uganda

**DOI:** 10.3389/fpls.2022.808597

**Published:** 2022-03-04

**Authors:** Julius J. Okello, Jolien Swanckaert, Daniel Martin-Collado, Bruno Santos, Benard Yada, Robert O. M. Mwanga, Anouk Schurink, Michael Quinn, Graham Thiele, Simon Heck, Timothy J. Byrne, Guy G. Hareau, Hugo Campos

**Affiliations:** ^1^International Potato Centre, Kampala, Uganda; ^2^Aardevo, Nagele, Netherlands; ^3^Aragon Agrifood Research and Technology Center [Centro de Investigación y Tecnología Agroalimentaria de Aragón (CITA)], Zaragoza, Spain; ^4^AbacusBio Ltd, Dunedin, New Zealand; ^5^National Crops Resources Research Institute, Kampala, Uganda; ^6^International Maize and Wheat Improvement Center, Texcoco, Mexico; ^7^International Potato Center, Lima, Peru; ^8^International Potato Center, Nairobi, Kenya; ^9^AbacusBio International Ltd., Edinburgh, United Kingdom

**Keywords:** crop breeding, trait ranking, economic incentives, sweetpotato, Uganda

## Abstract

Crop breeding programs must accelerate crop improvement, spur widespread adoption of new varieties and increase variety turnover they are to meet the diverse needs of their clients. More comprehensive quantitative approaches are needed to better inform breeding programs about the preferred traits among farmers and other actors. However, the ability of current breeding programs to meet the demands of their clients is limited by the lack of insights about value chain actor preference for individual or packages of traits. Ranking traits based on monetary incentives, rather than subjective values, represents a more comprehensive, consistent, and quantitative approach to inform breeding programs. We conducted a large pilot in Uganda to assess the implementation of a novel approach to trait ranking, using a uniquely large sample of diverse sweetpotato value chain actors. We found meaningful differences in trait ranking and heterogeneity among different actors using this approach. We also show our approach’s effectiveness at uncovering unmet demand for root quality traits and at characterizing the substantial trait demand heterogeneity among value chain players. Implementing this approach more broadly for sweetpotato and other crops would increase the effectiveness of breeding programs to improve food security in developing countries.

## Introduction

In response to demands of rapid social, demographic, climate and nutrition changes and gender inequality, the Consortium of International Agricultural Research Centers (CGIAR^1^) has embarked on an ambitious effort to increase impact of breeding programs across major staple crops.^2^ This is intended to help countries adapt to changes in distribution, meet food quality requirements, and contribute to the United Nations’ Sustainable Development Goal #2 of zero hunger by 2030. The CGIAR carries out public international breeding programs that are developing improved varieties for the benefit of smallholder farmers. These programs typically follow iterative cycles of creating relevant genetic variation, directional selection of superior genotypes, and thorough field testing in target populations of environments across which future varieties will be grown.

Since the 1960s, most crop breeding programs have focused on yield improvement and yield protecting traits. This work has resulted in increased adoption of improved varieties across the developing world, including in several locations in Asia (Estudillo and Otsuka, 2006), for example in Andhra Pradesh, India where adoption of improved chickpea varieties increased by well over 90% (Gumma et al., 2016). Nonetheless, changes in dietary preferences have increased the demand for quality-related traits in rice, resulting in discouragingly low adoption rates and low varietal turnover (Nayak et al., 2022), especially in more diverse and marginal environments of Africa (Kosmowski et al., 2020). More generally, breeding programs in root, tuber and banana crops have not evolved rapidly enough to respond to market needs (Thiele et al., 2020).

The concepts of participatory plant breeding (PPB) and participatory variety selection (PVS) were introduced in the 1970s and 1980s in many crop breeding programs and these, or similar approaches are currently being used to assess varietal preferences (Gibson et al., 2008) to incorporate client voices in variety development and accelerate variety replacement (Chambers and Ghildyal, 1985; Singh et al., 2020) for some crops. However, both PPB and PVS have three shortcomings: (i) continued dominance of breeders’ preferences in early selection processes; (ii) reliance on information of questionable comprehensiveness (Gibson et al., 2008); and (iii) a focus on farm/farmers as both producers and consumers thus neglecting the needs of urban consumers (De Janvry and Sadoulet, 2006; Custodio et al., 2016; Baffoe and Matsuda, 2017). Similarly, other important value chain actors (especially traders, processors, end consumers, and farmers from other geographical locations play multiple roles in the value chain) have been generally neglected. While breeding is best left to trained scientists, failure to make serious efforts to understand what should be developed for maximum impact and adoption undermines progress.

As a possible remedy to these shortcomings, and in contexts where PPB and PVS are still the main methods, we present a novel, comprehensive and quantitative approach for crop breeding programs to target key value chain actors (and nodes) more efficiently. Even in cases where programs have evolved beyond PPB and PVS, there is still inadequate understanding of the combination of preferred traits to target for adoption and maximum impact. This paper presents an alternative taking inspiration from an approach recently developed for animal breeding (Martin-Collado, 2015), and aims to describe the preferences of those actors, pinpoint the traits most desired, and create economic values for those traits. Our approach uses market intelligence to generate quantitative breeding indices to rank traits based on their monetary values, considering preferences from all potential breeding clients, from the farm to the consumer’s table. Thus, our analysis is less subjective in determining trait improvement priorities and we expect this information to impact and accelerate the adoption of novel varieties.

While the Green Revolution successfully catalysed adoption of improved varieties in smallholder farms in Asia, and subsequently other regions by relying on agronomic traits (Estudillo and Otsuka, 2006), the need for quality traits (namely, taste, flavor and texture) is now widely acknowledged (Kosmowski et al., 2020). To date, the processes for selecting breeding objectives and traits to be targeted in the genetic improvement of sweetpotato (*Ipomoea batatas*) and many other crops has drawn on PPB, PVS and farmer surveys. PPB was devised with intention for “bringing farmers back into breeding” (Almekinders et al., 2006; Ceccarelli, 2009) and for increasing farmer involvement earlier in the breeding process, hence early adoption (Ceccarelli, 2015). Participatory plant breeding typically occurs with heterogenous populations or where there are high levels of genetic diversity in the early stages of development. PVS, on the other hand, involves late engagement of farmers for selection toward the tail of the process when near-final breeding materials/clones are ready to be released (Witcombe et al., 1996; Walker, 2006; Shelton and Tracy, 2016). It relies on trait selection principles developed by Smith (1936) and Hazel (1943) in which multiple traits are selected at the same time using subjective ranking. PVS has allowed farmers to participate in adaptive testing/trials (Witcombe et al., 1996; Sperling et al., 2001) and has been widely used by plant breeding programs to capture farmer opinions. Farmer surveys have been used in breeding to obtain feedback from smallholder farmers on already-released varieties and have been applied by breeding program since 1970s (Bashaasha et al., 1995; Walker, 2006; Asrat et al., 2010; De Young et al., 2017; Ying et al., 2019). For purposes of our discussion, we note that all these approaches have focused predominantly on the farmer.

To maximize impact and accelerate not only genetic gains, but also actual variety adoption, breeding programs have to gain a much more granular view of the needs of actors beyond the farm to ensure that released varieties exhibit traits preferred by all key value chain actors and stakeholders. Thus, crop breeding should embrace a customer- and data-driven approach that creates value by addressing the actual needs of a diverse set of customers (Custodio et al., 2016). Doing so offers the potential for plant breeding to contribute to achieving broader societal goals such as reduced poverty and improved social inclusion, nutrition and health, climate resilience and environmental stewardship (Atlin et al., 2017). More generally, a tenet of successful innovation states that customers often struggle to clearly articulate their needs (Campos, 2021). Thus, breeding programs must gather information systematically from all potential “customers” of improved varieties.

**FIGURE 1:**
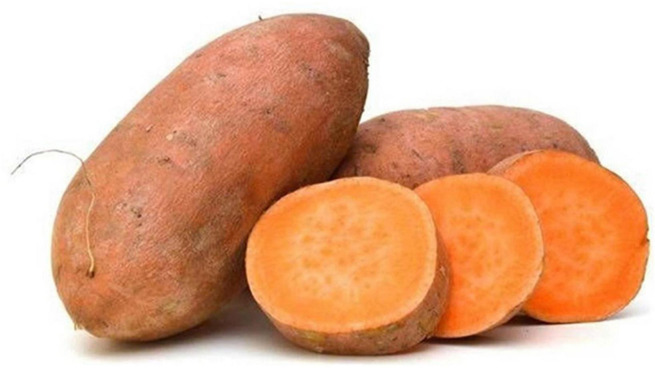
Picture of raw sweetpotato intact storage roots and cut inside surfaces: most households consume steamed roots.

We piloted our approach with 1,333 sweetpotato value chain actors in Uganda, a uniquely large and comprehensive sample size. In SSA, sweetpotato is a main crop and a staple food providing nutrition security to millions of smallholder farming households, especially through orange-fleshed sweetpotato (OFSP) varieties enriched with beta-carotene. Uganda is a leading sweetpotato producer in SSA, with a large per capita sweetpotato consumption of approximately 83 kg/year (Yada et al., 2011). Furthermore, Uganda is a secondary center of diversity for sweetpotato varieties (Yada et al., 2011). The sweetpotato storage root (Figure 1) is the primary product, consumed mostly as steamed roots, although some villagers consume the leaves as a vegetable. Thirty percent of households use the roots to make dry-processed products such as dried chips and flour (Engoru et al., 2005; Echodu et al., 2019). Approximately 80% of the sweetpotato farmers are smallholders (Shee et al., 2019) (less than one acre of land for sweetpotato) while about 53% of producers participate in the market. Nationally, 13.9% of sweetpotato roots are sold at the farm gate level, 42.6% at roadside markets, 35.6% in rural markets, and 7.9% in urban markets (Engoru et al., 2005). Multiple market actors including wholesalers, retailers, aggregators, and transporters trade in sweetpotato roots and processed products. Hence, Uganda provides a suitable location to pilot the approach.

## Materials and Methods

In this study, we used an **economic selection index approach** that provides a comprehensive and systematic approach to inform crop breeding programs about the most desired traits. The trait weightings derived from this method (Martin-Collado, 2015)—represented in monetary units—then determined the emphasis on specific traits during selection of a new variety. The monetary units infer a marginal economic value (profit) for a change in a specific trait unit, which is driven by associated costs and revenues related to the specific trait. Importantly, the underlying calculation method integrates both economic (e.g., prices and values) and non-market (socio-demographic) drivers in ranking traits. Candidate varieties were subsequently ranked, based on estimates of genetic merit combined with these trait rankings, in an economic selection index (see Section “Materials and Methods”).

As discussed further in the Methodology section, the approach used was as follows. First, information was gathered through extensive stakeholder and community consultation, key informant interviews, and an extensive desk literature review. Second, focus group discussions were used to design a survey that systematically captured the data from sampled value chain actors at different nodes. Third, resultant survey data were used to derive clusters/typologies of actors to generate breeding selection indices from trait monetary equivalences that had been established by a multistakeholder team of breeders, social and gender scientists, seed producers, root producers, transporters, retailers, processors, and consumers. The approach used the **P**otentially **A**ll **P**airwise **R**an**K**ings of all Possible **A**lternatives algorithm, based on pairwise co-joint analysis (Bryne et al., 2012).

### Data Sources and Generation Process

Data was collected in Uganda from primary and secondary sources. The secondary data were obtained from a systematic review of existing literature. Primary sources included: (i) a workshop with value chain actors organized as a series of focus group discussions (FGDs) targeting seed producers and multipliers, root producers, traders (retailers and wholesalers), processors, and consumers; (ii) community forums with seed multipliers; (iii) household surveys; and (iv) market surveys. The workshop, FGDs and community forums generated understandings about the most desired traits, the desired direction of improvement, gender differences in trait preferences, and price differences across a range of variation at the trait level by monetizing the traits.

Household surveys targeted a large sample of root producers (*n* = 1,000) who were randomly selected from sweetpotato farmers in the central Uganda region, with 984 successfully completing the survey. The sample was stratified by gender, and the surveys captured sociodemographic information on respondents (e.g., age, sex, education, farm size and type, production orientation) and their value chain activities. We also surveyed 52 sweetpotato seed multipliers. The market survey targeted sweetpotato traders (*n* = 134) and consumers (*n* = 147) drawn from rural and urban sweetpotato retail markets through systematic random sampling. Data were collected using an information and communication technology-based platform (ICT) and analysed using the 1000minds software to generate breeding indices. The indices formed the basis for ranking specific traits relative to each other.

### Analytical Framework for the Economic Selection Index Approach

The analysis was conducted separately for both sociodemographic and 1000minds^®^ preference surveys and then jointly to provide a better understanding of the heterogeneity of responses. The 1000minds technique is a special case of conjoint analysis that uses pairwise comparison of traits, thus substantially reducing the cognitive burden on the respondents. Further details are provided in Martin-Collado (2015). Principal component analysis (PCA) and cluster analysis (CA) were used to create homogeneous groups (i.e., clusters) of respondents in terms of trait preferences. Statistical analysis and tests were carried out in R software. Specifically, PCA was performed using the *pca* procedure in the *FactoMineR* package in R software, based on ranks for the 13 traits (color was considered as a single trait and excluded the trait “price per 100 kg bag”) in the 1000minds^®^ preference survey. We use CA, on the other hand, to identify groups of respondents who have similar trait preference patterns without making any *a priori* assumptions as to which groups are likely to have different preferences.

The calculation of economic values from preference data enables the development of economic selection indices to rank varieties. Trait economic values were calculated according to the preference (%) for each trait relative to the preference (%) for the trait expressed in monetary terms in the survey, “price per 100 kg bag” (Byrne et al., 2011). Thus, the economic value per trait unit was calculated to reflect a unit change in the trait, expressed mathematically as:


(1) $${\text{pEV}}_{\text{qi}}=\left[\frac{P_{i\,q}}{\alpha_{i}}\right]\times \beta_{q},$$

where for trait *i* and individual respondent *q*, *P* is the preference (%) for each trait, α is the number of units represented in the trait level (to convert to the desired final trait unit), and β is the monetary value per preference (%) for individual *q*. Values for β were calculated as:


(2) $$\beta_{q}=\left[\frac{a\,m\,v}{P\,m\,v_{q}}\right],$$

where α*mv* is the number of units represented in the level for the monetary trait (Uganda shillings, UGX 5,000: One United States Dollar was equal 3, 650 UGX at the time of the study) and, for individual respondent *q*, *Pmv* is the preference (%) for the monetary trait. An example of the calculation is provided in the Findings section.

The economic value of a trait in breeding program units (*EV*_*BP*_) was calculated by multiplying the economic value of a trait in survey units (*EV*_*S*_) by the unit transformation (*ut*), as follows:


(3) $$E\,V_{B\,P}=E\,V_{S}\times u\,t,$$

The *EV*_*BP*_ represent the weights that would be applied to estimates of genetic merit (either breeding values or trait means), to rank varieties on the selection index. For the case of root size, the unit of root size in the survey was the percentage (1%) of roots that are of medium size, whereas the unit of root size in the breeding program was a score (1 to 9, excellent to terrible). Hence, an increase of 1 score, therefore, resembled a decrease of 12.5% in roots of medium size (100% / 8), assuming that medium roots are considered excellent on the 1 to 9 scale. For all respondents, the economic value for root size in breeding program units (*EV*_*BP*_) therefore equals UGX 5,338 per score [= UGX 427 × 12.5(%)]. Note that breeding values presented in this pilot were computed based on several assumptions (see Supplementary Table 1) about the relationship between survey units and breeding program units for each trait and are illustrative.

Lastly, relative emphasis (*R*) was on each trait (*t*) in the selection index, and therefore the potential to make genetic progress. The genetic standard deviations were used in the following equation:


(4) $$R_{t}=\left(E\,V_{B\,P\,t}\times \sigma_{a\,t}\right)/\sum_{t=1}^{n}\left(E\,V_{B\,P\,t}\times \sigma_{a\,t}\right),$$

where, σ_*a*_ is calculated from the square root of genetic variance for genotypes $\left(\sqrt{\sigma_{G}^{2}}\right)$, and all other parameters are described in Equation (3). The relative emphasis (*R*) on each trait (*t*) in the selection index has also been converted to relative emphasis in UGX per USh100 of index progress by multiplying *R* for trait *t* by 100. Supplementary Table 1 provides information on the parameters used in the analysis.

## Results

**FIGURE 2:**
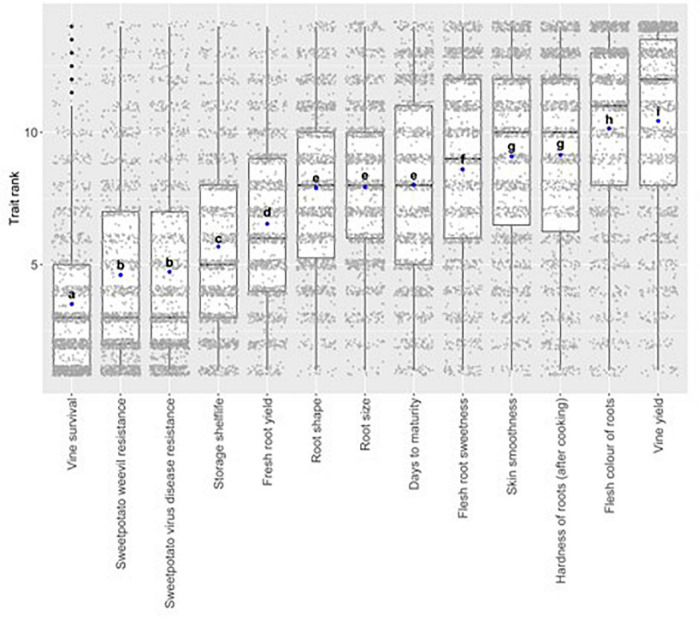
Trait preference^1^ ranks of 13 sweetpotato traits included in the lOOOMinds^®^ survey used to generate economic selection index in Uganda (for all respondents). Each gray dot represents an individual answer (respondent preference for each trait) and the blue doc represents the average preference rank of a trait, ordered from most (left) to least prefened Different letter- denote significant (p-value < 0.05) differences between the traits.

Sweetpotato value chain actors attach importance to a wide range of traits, ranging from agronomic production to quality varying according to their position in the value chain (Figure 2). Vine survival (crucial as sweetpotato planting material), weevil resistance and sweetpotato virus disease (SPVD) resistance are the most preferred traits. The distribution of responses (gray dots) highlights the variation in preferences among respondents within traits and indicates that trait preferences were strongly heterogeneous. The heterogeneity is substantial, even for the traits that are ranked low, on average—though this could be driven by location in the value chain.

**FIGURE 3:**
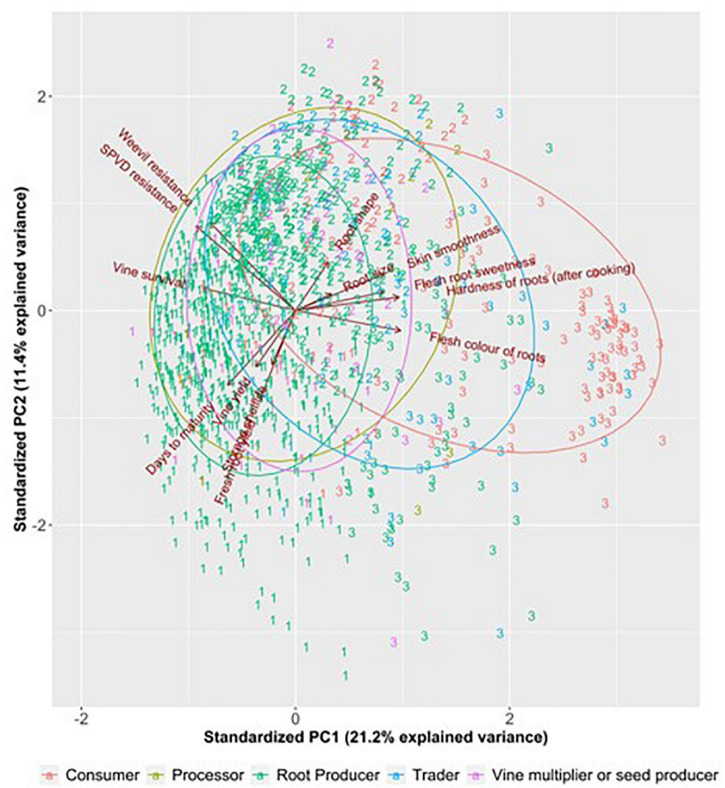
Principal component analysis (PCA) plot separating respondents based on actor type and their preference for sweetpotato traits in Uganda. The numbers represent the group the respondent was assigned to (1 = “productive output” group. 2 = “plant robustness” group, and 3 = “root quality” group); Dry matter = hardness of roots after cooking, flesh color = flesh color of roots, maturin’ time = days to maturin’, shelflife = storage shelflife, SPVD resistance = sweetpotato virus disease resistance, sweetness = flesh sweetness, weevil resistance = sweet potato weevil resistance.

Multivariate analysis of preferences using principal component analysis and cluster analysis (Figure 3 and Table 1) identified three broad clusters of actors: (i) actors who preferred short maturity, high fresh root yield, high vine yield, and longer storage shelf life—the “productive output” cluster; (ii) actors that preferred resistance to SPVD and sweetpotato weevil—the “plant robustness” cluster; and (iii) actors prioritized root flesh color, hardness/firmness of roots after cooking, flesh sweetness, skin smoothness and specific root size—the “root quality” cluster. Note that clusters are not uniquely separate from each other but that there is a continuum of preferences, implying that there are many respondents with intermediate positions between the preferences of any clusters.

**TABLE 1:** Average trait preference ranks of sweetpotato traits by value chain actors in Uganda, by cluster^1^ of value chain actors.

	Average preference rank					
Trait	Across groups	Productive output (*n* = 686)	Plant robustness (*n* = 450)	Root quality (*n* = 187)	P-value
Vine survival	3.50	2.99^a^	3.06^a^	6.48^b^	< 2 × 10^–16^
Sweet potato Weevil resistance	4.61	4.31^a^	3.15^b^	9.23^c^	< 2 × 10^–16^
Sweet potato Virus Disease resistance	4.73	4.27^a^	3.09^b^	10.32^c^	< 2 × 10^–16^
Storage shelf life	5.67	5.06^a^	6.56^b^	5.85^c^	6.1 × 10^–14^
Fresh root yield	6.54	5.61^a^	7.81^b^	6.91^c^	< 2 × 10^–16^
Root shape	7.90	8.38^a^	7.53^b^	7.02^b^	7.4 × 10^–9^
Root size	7.92	8.22^a^	7.98^a^	6.76^b^	6.3 × 10^–8^
Days to maturity	8.01	5.86^a^	10.46^b^	10.05^b^	< 2 × 10^–16^
Flesh sweetness	8.61	10.22^a^	7.63^b^	4.96^c^	< 2 × 10^–16^
Skin smoothness	9.09	10.51^a^	8.29^b^	5.83^c^	< 2 × 10^–16^
Hardness of roots after cooking	9.15	10.87^a^	8.28^b^	4.90^c^	< 2 × 10^–16^
Flesh color of roots	10.14	11.43^a^	10.08^b^	5.57^c^	< 2 × 10^–16^
Vine yield	10.42	9.44^a^	11.60^b^	11.24^b^	< 2 × 10^–16^

*^1^Preference ranks are sorted from most (first row) to least preferred across groups, where a lower rank means the trait is more preferred. The traits that differ significantly between group(s) are either highlighted green (= most preferred) or red (= least preferred). Thus, the cluster group of respondents preferring root quality traits preferred vine survival significantly less compared to the other two cluster groups. Different letters indicate statistically significant (p-value < 0.05) differences between the group(s).*

As it might be expected, most consumers fell within the “root quality” cluster, while traders/retailers and processors were in an intermediate position between producers (“productive output”) and consumers (Figure 3).

Table 2 summarizes respondents’ characteristics by cluster and shows differences in trait rankings by actor type. Note the large representation of root producers (*n* = 984), which was selected by design because this actor group is proportionally larger compared to other groups in the value chain. Root producers are also the most immediate customers and some of them interact directly with genetic improvement programs.

**TABLE 2:** Variables that explain differences in ranking between cluster groups of sweetpotato value chain actors in Uganda.

Variable	Definition	Productive output	Plant robustness	Root quality	p-value*
Actor type	Consumer (%, *n* = 157)	15	32	53	< 0.001
	Processor (%, *n* = 17)	41	47	12	
	Root producer (%, *n* = 984)	61	32	7	
	Trader (%, *n* = 94)	23	45	32	
	Vine multiplier (%, *n* = 68)	41	50	9	

*P-values are from the Fisher’s Exact Test for count data (fisher.test function in R software) calculated across the clusters.

### Economic Values of Sweetpotato Traits

Table 3 presents the economic/monetary values of the different traits. The first three columns present the economic values of traits in survey units (EV_*s*_), unit transformation (*ut*) coefficients, and in breeding program units (*EV*_*BP*_). The *EV*_*BP*_ weights per cluster are presented in the last four columns. The negative values are a result of the way traits are defined in the breeding program. For instance, root size in the breeding program was a score on a 1–9 scale (excellent to terrible) while the units presented in the survey were based on increases in regular/good shape of roots. As such, the direction of the unit transformation factor converts the survey units to the breeding program units for each trait.

**TABLE 3:** Economic values of traits in survey units (*EV*_*S*_), unit transformation factors (*ut*), and in breeding program units (*EV*_*BP*_) generated from the economic selection index approach for sweetpotato value chain actors in Uganda.

Trait			Selection index			
	EV_S_	ut	EV_BP_ Overall sample	EV_BP_ productive output index	EV_BP_ plant robustness index	EV_BP_ root quality index
Vine survival (%)	+2,285	+1.00	+2,285	+19,183	+26,196	+22,371
Sweetpotato weevil resistance (1–9 score)	–2,078	+12.50	–25,975	–5,687	–8,842	–6,015
Sweetpotato virus disease resistance (%)	–2,043	+1.00	–2,043	–1,901	–2,912	–1,435
Storage shelf life (week)	+1,111	+7.00	+7,777	+6,594	+8,008	+11,480
Fresh root yield (tons/ha)	+5,748	+4.05	+23,261	+64,157	+71,658	+105,394
Root shape (1–9 score)	+419	–12.50	–5,238	–2,384	–3,674	–5,573
Storage root size (1–9 score)	+427	–12.50	–5,338	–2,631	–3,955	–6,408
Days to maturity (days)	–1,165	+1.00	–1,165	–3,737	–2,734	–3,194
Flesh sweetness (1–9 score)	+10,661	+0.44	+4,738	+3,570	+7,702	+14,495
Skin smoothness^1^	+9,595	-	-	-	-	-
Hardness of roots after cooking (1–9 score)	+10,143	+0.50	+5,072	+3,111	+6,999	+15,305
Flesh color of roots (1–30 score)	+8,578	+0.14	+1,183	+4,160	+8,245	+21,492
Vine yield (tons/ha)	+835	+13.49	+11,264	+124,729	+111,766	+121,416

*^1^Breeding program trait definition/units was not available, hence not included in the calculations.*

**FIGURE 4:**
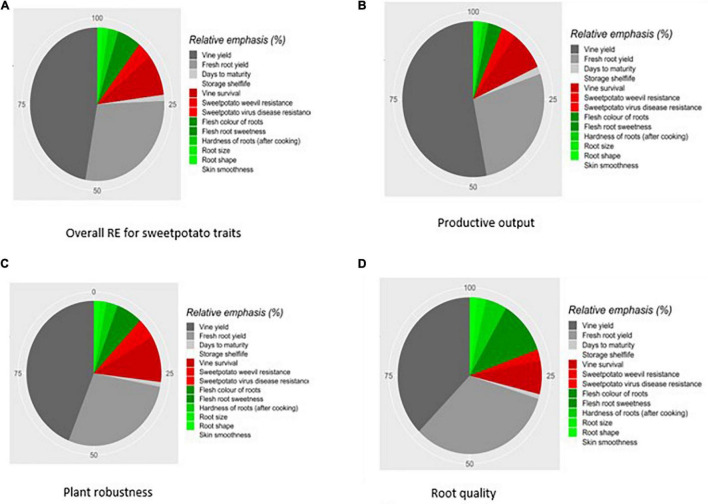
Relative emphasis (RE) of sweetpotato traits from the application of economic selection approach using survey data collected from value chain actors in Uganda. **(A)** Overall RE for sweetpotato traits. **(B)** Productive output. **(C)** Plant robustness. **(D)** Root quality. Traits in this panels **(A–D)** legends are ordered from highest to lowest contribution to genetic progress within each category of traits. Production traits are labeled in diminishing gray, according to their contribution. Plant robustness traits are labeled in diminishing red, and root quality traits in diminishing green. Data on skin smoothness and storage shelf life was missing and hence this trait was dropped.

Figures 4A–D present the relative emphasis (RE) of traits in the selection indices for the three clusters, which is a measure of the importance attached to a trait, by combining estimates of genetic variation with *EV*_*BP*_. Figure 4A indicates that vine yield, fresh root yield, and vine survival dominated other traits in the productive output group, in part because those traits have high genetic standard deviations. Differences in trait emphasis between clusters is apparent when comparing Figures 4B–D.

To understand how selection indices align with the preferences of the cluster groups, REs were added together within the cluster (Table 4). Vine and root yield were clearly ranked highest, indicating that production traits continue to dominate other traits.

**TABLE 4:** Relative emphasis (%) on production, plant robustness, and root quality for the overall index and the productive output, plant robustness, and root quality indices generated from the economic selection index approach.

Relative emphasis (%) per trait category	Overall index	Productive output index	Plant robustness index	Root quality index
Productive output	77.0	**81.9**	73.1	71.2
Plant robustness	12.2	11.4	**14.9**	9.2
Root quality	10.8	6.7	12.0	**19.6**

*Bold numbers denote highest relative emphasis in percentage terms.*

## Discussion

Sweetpotato breeding programs in Uganda and other developing countries continue to be focused on storage root yields and, more recently, nutrition objectives that target biofortification of staple crops. Traits relevant to other value chain actors have not found their way into the breeding programs on a regular basis, despite evidence that neglect of quality traits (Thiele et al., 2020) impedes adoption. PPB, PVS and surveys used for trait selection have primarily targeted farmers (Bashaasha et al., 1995; Gibson et al., 2011) and, in some cases, generated non-comprehensive information (Gibson et al., 2011), which leads to a disconnect between the traits pursued by the breeding program and what clients desire (Teeken et al., 2018). However, it is important to note that low adoption of new varieties may also be attributed to the lack of effective seed delivery systems and weak extension systems. Such misalignments can undermine nutrition and/or food security for farm households by limiting access to most preferred varieties or by breeding outputs failing to deliver the package of traits required for both adoption and impact. Changes in trait preferences are expected to accompany improvements in socioeconomic status of clients of breeding programs, notably the consumers. For breeders, this factor requires intimate knowledge of current and future markets as client needs change over time.

This case study presents a novel approach to setting objectives for crop breeding, designed to help prioritize traits for different nodes of the value chain using comprehensive market insights. This approach was used for sweetpotato in our study and with some improvements may be extended to other sweetpotato breeding programs and crops. It informs the definition of breeding objectives as breeders often face the difficulty of balancing the selection of a few preferred traits from a wide array of “nice-to-have” traits, some of which can be negatively correlated (have trade-offs), and have multidimensional (Martin-Collado, 2015; Gemenet et al., 2020) preferences. The new approach clearly demonstrates that beneficiaries of breeding programs across the value chain have heterogeneous preferences that go beyond high yields on the farm.

Overall, producers prioritized fresh root yield below vine survival, sweetpotato weevil and SPVD resistance, and storage shelf life. But we also found significant variations in preferences for these traits within this actor group, implying that there is no one-size-fits-all scenario and that new varieties with different combinations of traits can target different producer types.

Surprisingly, the sensory/quality traits—namely, flesh color, root flesh sweetness, hardness/firmness of the roots after cooking—were ranked very low contrary to recent suggestions in the literature (Mwanga et al., 2020). However, this finding should be treated with caution given the design limitations of the new approach (which we turn to in a moment). Vitamin A enhancement ranked low and this finding suggests that while consumers may have a need for more vitamin A in their diets, they may not demand this trait because they lack understanding about its contribution to health, and about the relationship between orange color and high vitamin A content in the roots. Traders expressed a strong preference for root shape, likely because it is an important aspect for packing and transporting.

In summary, the key contribution of this new approach is that traits are prioritized using economic values derived from systematic market intelligence, rather than subjective values, and targets all key value chain actors. Our study also underscores the relevance of gathering market insights from multiple actors in the value chain in defining breeding objectives. To gain sufficient insight about markets served, and to articulate their trait preferences, and traits required for impact and to achieve SDGs, it is essential to collect insights from other experts such as social and gender scientists, nutritionists, food technologists, economists, and marketing specialists, as well as plant breeders. It would be counterproductive, and unfair, for plant breeders to assume such roles since their training, experience and interests are best put to good use by increasing genetic gains and focusing on genetic research to develop varieties able to deliver climate resilience, food security and aligned to rapidly evolving social landscapes.

### Limitations of the New Approach

While this paper represents substantial progress in estimating the economic/monetary worth of crop traits in a manner that can better inform breeding programs, we acknowledge three main remaining challenges. First, without understating the significantly increasing complexity in variety design and deployment, the current **economic selection index** does not differentiate the types of questions administered to different value chain actors. Thus, traders and consumers ranked and valued production traits that may not be directly economically relevant to the entire value chain. Second, due to low literacy, it was quite challenging to describe traits in terms that were understood by all respondents the same way, and that could be linked to breeding program definitions. For example, scores of beta-carotene content of roots (a proxy for vitamin A richness) were hard to translate into a language that accurately captures the value of this trait in ways most study respondents could understand and relate to because of low literacy levels. Breeders use scores based on the intensity of orange color for this trait, and most respondents would not understand the relationship between the color and beta-carotene content. Third, incomplete phenotyping information for the national breeding program in our study (Uganda) made it difficult to create economic weights for all the traits as described in the methods section. These methodological/software challenges pinpoint areas where further refinement of the new approach is needed for wider applicability.

The first such refinement will be to build a framework/logic into the approach that allows different traits or trait combinations to be administered to different actors based on the value chain nodes they represent. The second refinement calls for the need of more studies to ground-truth trait selection and breeding lexicons with nutritionists and food scientists to ensure a clear description of traits for the target respondents. This study relied on information from expert and stakeholder consultation only because work by nutritionists in developing such lexicons was ongoing at the time of this study. The third refinement will balance out the survey population, which was almost 70% root producers in our case, thus weighting the findings in favor of root producers. Future application of this approach in a value chain context should assess if and how equal representation of the actors in the sample influences the results. These refinements are critical if this approach is to be scaled up to other crops and contexts.

## Conclusion

This study has demonstrated the merits of value chain-wide market intelligence to inform crop breeding programs in developing countries. Despite the limitations with our approach (acknowledged above), it presents a new and objective method for prioritizing traits in crop breeding programs with good insights from the market based on economic incentives instead of subjective ranking. We conclude from this pilot that to be successful in understanding actors’ preferences, it is essential to crowdsource market intelligence from a wide range of actors. We also note that trait preferences are not homogenous: we find distinct actor groups, even within a given value chain node, who have distinct preferences for specific combinations, or baskets, of traits. The large heterogeneity in trait preferences that exist within root producers as a cluster, and other actors, clearly indicates that the current approach to plant breeding which focuses mostly on farmers is not optimal. As expected, a consumer who does not grow the crop is likely to rank sensory quality traits highly while a farmer would express preference for yield and yield protecting traits such as drought tolerance and disease and pest resistance. We further conclude that subjective ranking of traits has tended to mask the real trade-offs that clients of the breeding program have to make when comparing traits. Notably, economic valuation and preference ranking in this pilot highlight, in general, the continued importance of production traits—vine survival, yield, and disease and pest resistance. However, there is also strong demand for root quality traits, which further suggests that breeding needs to be pluralistic. Lastly, our pilot demonstrates that real-time gathering and analysis of value chain-wide market intelligence using internet-based platforms designed for developed countries can be performed successfully for plant breeding purposes.

## Data Availability Statement

The raw data supporting the conclusions of this article will be made available by the authors, without undue reservation.

## Ethics Statement

Ethical review and approval was not required for the study on human participants in accordance with the local legislation and institutional requirements. The patients/participants provided their written informed consent to participate in this study.

## Author Contributions

JJO led the study design and implementation and writing of the manuscript and revisions. BS, TB, AS, and DM-C co-led the study design and implementation, data analysis, and review of the manuscript. JS contributed to data analysis and writing of the manuscript. BY and ROMM co-led the study design and reviewed several drafts of the manuscript. HC conceived the study, cowrote the manuscript, and reviewed several drafts. GT, SH, MQ, and GH reviewed and edited the drafts. All authors contributed to the article and approved the submitted version.

## Conflict of Interest

TB was employed by AbacusBio Ltd., United Kingdom. BS and AS were employed by the company AbacusBio Ltd., New Zealand. JS was employed by Aardevo Ltd. The remaining authors declare that the research was conducted in the absence of any commercial or financial relationships that could be construed as a potential conflict of interest.

## Publisher’s Note

All claims expressed in this article are solely those of the authors and do not necessarily represent those of their affiliated organizations, or those of the publisher, the editors and the reviewers. Any product that may be evaluated in this article, or claim that may be made by its manufacturer, is not guaranteed or endorsed by the publisher.

## Footnotes

1
https://www.cgiar.org

2
https://excellenceinbreeding.org/

## References

[B1] Almekinders C. Hardon J. Christinck A. Humphries S. Pelegrina D. Sthapit B. (2006). *Bringing Farmers Back Into Breeding. Experiences With Participatory Plant Breeding and Challenges for Institutionalization.* Wageningen: CTA CGSpace. 10.1080/1389224X.2013.764718

[B2] Asrat S. Yesuf M. Carlsson F. Wale E. (2010). Farmers’ preferences for crop variety traits: lessons for on-farm conservation and technology adoption. *Ecol. Econ.* 69 2394–2401. 10.1016/j.ecolecon.2010.07.006

[B3] Atlin G. N. Cairns J. E. Das B. (2017). Rapid breeding and varietal replacement are critical to adaptation of cropping systems in the developing world to climate change. *Glob. Food Secur.* 12 31–37. 10.1016/j.gfs.2017.01.008 28580238 PMC5439485

[B4] Baffoe G. Matsuda H. (2017). Why do rural communities do what they do in the context of livelihood activities? Exploring the livelihood priority and viability nexus. *Community Dev.* 48 715–734. 10.1080/15575330.2017.1366927

[B5] Bashaasha B. Mwanga R. O. M. Ocitti p’Obwoya C. Ewell P. T. (1995). *Sweetpotato in the Farming and Food Systems of Uganda: A Farm Survey Report.* Kampala: International Potato Center (CIP), Nairobi & National Agricultural Research Organization (NARO).

[B6] Bryne T. J. Amer P. R. Fennessy P. F. Hansen P. Wickham B. W. (2012). A preference-based approach to deriving breeding objectives: applied to sheep breeding. *Animal* 6 778–788. 10.1017/S1751731111002060 22558925

[B7] Byrne T. Fennessy P. Smith K. Hansen P. Am P. (2011). Preference-based approaches to deriving breeding objectives, application to sheep and plant breeding. *Proc. Assoc. Adv. Animal Br. Genet.* 19 35–38.

[B8] Campos H. (2021). *The Innovation Revolution in Agriculture.* Switzerland: Springer.

[B9] Ceccarelli S. (2009). Evolution, plant breeding and biodiversity. *J. Agric. Environ. Int. Dev.* 103 131–145. 10.12895/jaeid.20091/2.28

[B10] Ceccarelli S. (2015). Efficiency of plant breeding. *Curr. Sci.* 55 87–97.

[B11] Chambers R. Ghildyal B. P. (1985). Agricultural research for resource-poor farmers: the farmer-first-and-last model. *Agr. Admin.* 20 1–30. 10.1016/0309-586X(85)90063-9

[B12] Custodio M. C. Demont M. Laborte A. Ynion J. (2016). Improving food security in Asia through consumer-focused rice breeding. *Glob. Food Secur.* 9 19–28. 10.1016/j.gfs.2016.05.005

[B13] De Janvry A. Sadoulet E. (2006). “Progress in the modeling of rural households’ behavior under market failures,” in *Poverty, Inequality and Development*, eds De Janvry A. Kanbur R. (Boston, MA: Springer), 155–181. 10.1007/0-387-29748-0_9

[B14] De Young D. J. Reyes B. Osorno J. Mejia G. Villatoro J. C. Maredia M. K. (2017). *An Overview of Bean Production Practices, Varietal Preferences, and Consumption Patterns in The Milpa System of the Guatemalan Highlands: Results of a Farm Household Survey (No. 1099-2018-1004).* Available onlin at: https://ageconsearch.umn.edu/record/268951/ (accessed September 16, 2021).

[B15] Echodu R. Edema H. Wokorach G. Zawedde C. Otim G. Luambano N. (2019). Farmers’ practices and their knowledge of biotic constraints to sweetpotato production in East Africa. *Physiol. Mol. Plant Proc.* 105 3–16. 10.1016/j.pmpp.2018.07.004 31007371 PMC6472603

[B16] Engoru P. Mugisha J. Bashaasha B. (2005). An estimate of the contribution of local sweet potato value adding options of processing and storage to producer’s sweet potato gross margins in eastern Uganda. *Afr. Crop Sci. Conf. Proc.* 7 745–751.

[B17] Estudillo J. P. Otsuka K. (2006). Lessons from three decades of green revolution in the philippines. *Dev. Econ.* 44 123–148. 10.1111/j.1746-1049.2006.00010.x

[B18] Gemenet D. C. da Silva Pereira G. De Boeck B. Wood J. C. Mollinari M. Olukolu B. A. (2020). Quantitative trait loci and differential gene expression analysis reveal the genetic basis for negatively associated beta-carotene and starch content in hexaploidy sweetpotato [*Ipomoea batatas* (L.) lam.]. *Theor. Appl. Genet.* 133 23–36. 10.1007/s00122-019-03437-7 31595335 PMC6952332

[B19] Gibson R. W. Byamukama E. Mpembe I. Kayongo J. Mwanga R. O. M. (2008). Working with farmer groups in uganda to develop new sweet potato cultivars: decentralisation and building on traditional approaches. *Euphytica* 159 217–228. 10.1007/s10681-007-9477-4

[B20] Gibson R. W. Mpembe I. Mwanga R. O. M. (2011). Benefits of participatory plant breeding (PPB) as exemplified by the first-ever officially released PPB-bred sweet potato cultivar. *J. Agric. Sci.* 149 625–632. 10.1017/S0021859611000190

[B21] Gumma M. K. Charyulu Deevi K. Mohammed I. A. Varshney R. K. Gaur P. Whitbread A. M. (2016). Satellite imagery and household survey for tracking chickpea adoption in Andhra Pradesh. *India. Int. J. Red. Sen.* 37 1955–1972. 10.1080/01431161.2016.1165889

[B22] Hazel L. N. (1943). The genetic basis for constructing selection indexes. *Genetics* 28, 476–490. 10.1093/genetics/28.6.476 17247099 PMC1209225

[B23] Kosmowski F. Alemu S. Mallia P. Stevenson J. Macours K. (2020). *Shining A Brighter Light: Comprehensive Evidence on Adoption and Diffusion of CGIAR-Related Innovations in Ethiopia.* Rome: Standing Panel on Impact Assessment (SPIA).

[B24] Martin-Collado D. (2015). Analyzing the heterogeneity of farmers’ preferences for improvements in dairy cow traits using farmer typologies. *J. Dairy Sci.* 98 4148–4416. 10.3168/jds.2014-9194 25864048

[B25] Mwanga R. O. M. Mayanja S. Swanckaert J. Nakitto M. Zum Felde T. Grüneberg W. (2020). Development of a food product profile for boiled and steamed sweetpotato in uganda for effective breeding. *Int. J. Food Sci. Technol.* 56 1385–1398. 10.1111/ijfs.14792 33776240 PMC7983908

[B26] Nayak S. Hossain M. Das K. (2022). *Market-Driven Varietal Testing and Positioning in Seed Chain. A Blog.* International Rice Research Institute. Available online at: https://rbi.irri.org/market-driven-varietal-testing-and-positioning-in-seed-chain (accessed January 10, 2022).

[B27] Shee A. Mayanja S. Simba E. Stathers T. Bechoff A. Bennett B. (2019). Determinants of postharvest losses along smallholder producers maize and sweetpotato value chains: an ordered probit analysis. *Food Sec.* 11 1101–1120. 10.1007/s12571-019-00949-4

[B28] Shelton A. C. Tracy W. F. (2016). Participatory plant breeding and organic agriculture: a synergistic model for organic variety development in the United States. *Elementa Sci. Anthrop.* 4:000143. 10.12952/journal.elementa.000143

[B29] Singh R. P. Chintagunta A. D. Agarwal D. K. Kureel R. S. Kumar S. J. (2020). Varietal replacement rate: prospects and challenges for global food security. *Glob. Food Secur.* 25:100324. 10.1016/j.gfs.2019.100324

[B30] Smith F. H. (1936). A discriminant function for plant selection. *Ann. Eugen.* 7 240–250.

[B31] Sperling L. Ashby J. A. Smith M. E. Weltzien E. McGuire S. (2001). A framework for analyzing participatory plant breeding approaches and results. *Euphytica* 122 439–450. 10.1023/A:1017505323730

[B32] Teeken B. Olaosebikan O. Haleegoah J. Oladejo E. Madu T. Bello A. (2018). Cassava trait preferences of men and women farmers in nigeria: implications for breeding. *Econ. Bot.* 72 263–277. 10.1007/s12231-018-9421-7 30573920 PMC6267705

[B33] Thiele G. Dufour D. Vernier P. Mwanga R. O. M. Parker M. L. Schulte Geldermann E. (2020). A review of varietal change in roots, tubers and bananas: consumer preferences and other drivers of adoption and implications for breeding. *Int. J. Food Sci. Technol.* 56 1076–1092. 10.1111/ijfs.14684 33776222 PMC7983933

[B34] Walker T. S. (2006). *Participatory Varietal Selection, Participatory Plant Breeding, and Varietal Change.* Availble online at: https://openknowledge.worldbank.org/handle/10986/9182 (accessed September 15, 2021).

[B35] Witcombe J. R. Joshi A. Joshi K. D. Sthapit B. R. (1996). Farmer participatory crop improvement. I. varietal selection and breeding methods and their impact on biodiversity. *Exp. Agric.* 32 445–460. 10.1017/S0014479700001526

[B36] Yada B. Tukamuhabwa P. Alajo A. Mwanga R. O. M. (2011). Field evaluation of ugandan sweetpotato germplasm for yield, dry matter and disease resistance. *Sci. Afr. J. Plant Soil.* 28 142–146. 10.1080/02571862.2011.10640026

[B37] Ying H. Yin Y. Zheng H. Wang Y. Zhang Q. Xue Y. (2019). Newer and select maize, wheat, and rice varieties can help mitigate N footprint while producing more grain. *Glob. Change Biol.* 25 4273–4281. 10.1111/gcb.14798 31418955

