# Supplementary material for: Market Intelligence and Incentive-Based Trait Ranking for Plant Breeding: A Sweetpotato Pilot in Uganda
Source: Front Plant Sci. 2022 Mar 4;13:808597. doi: 10.3389/fpls.2022.808597 (PMC8934386; doi:10.3389/fpls.2022.808597)
Supplement: Supplementary file 1 [file Table_1.docx]

**Supplementary Table 1**: Assumptions, units, valuation method, trait level change, and value of change (Ugandan shillings, UGX) per trait level change, for traits included in the 1000Minds preference survey

|  | **Survey** | **Breeding program** |
| --- | --- | --- |
| *Trait* | Root size | Root size |
| *Definition* | Percentage of roots of medium size (score 5)  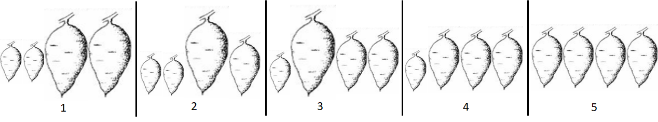 | Overall assessment of storage root size based on inspection of the harvested roots (on a scale from 1 to 9) |
| *Unit* | 1% | 1 score |
| *Matching units* | 100.0% (all roots of preferred size) | 1 (excellent) |
|  | 87.5% | 2 |
|  | 75.0% | 3 (good) |
|  | 62.5% | 4 |
|  | 50.0% | 5 (fair) |
|  | 37.5% | 6 |
|  | 25.0% | 7 (poor) |
|  | 12.5% | 8 |
|  | 0.0% (no roots of preferred size) | 9 (terrible) |
| *Transforming breeding program* $\sigma_{a}$ *to survey unit* | 12.50%  8.49% | 1 score  0.68 score (=$\sigma_{a}$) |
| *Assumptions* | Medium size is the preferred (= excellent) storage root size.  The correlation between the two units is assumed to be 1. | |
|  | **Survey** | **Breeding program** |
| *Trait* | Fresh root yield | Storage root yield |
| *Definition* | Number of 100 kg-bags of fresh roots per acre | Storage root yield in tonnes per hectare |
| *Unit* | One 100 kg-bag per acre | Tonnes per hectare |
| *Matching units* | 2.47 acre | 1 hectare |
|  | 1,000 kg | 1 tonne |
| *Transforming breeding program* $\sigma_{a}$ *to survey unit* | 4.05 100 kg-bags per acre (= ((1/2.47) x 1,000) / 100)  12.65 100 kg-bags per acre | 1 tonne per hectare  3.13 tonnes per hectare (= $\sigma_{a}$) |
| *Assumptions* | None | |
|  | **Survey** | **Breeding program** |
| *Trait* | Vine yield | Foliage yield |
| *Definition* | Number of 30 kg-bags of vines per acre | Foliage yield in tonnes per hectare |
| *Unit* | One 30 kg-bag per acre | Tonnes per hectare |
| *Matching units* | 2.47 acre | 1 hectare |
|  | 1,000 kg | 1 tonne |
| *Transforming breeding program* $\sigma_{a}$ *to survey unit* | 13.49 30 kg-bags per acre (= ((1/2.47) x 1,000) / 30)  144.03 30 kg-bags per acre | 1 tonne per hectare  10.68 tonnes per hectare (= $\sigma_{a}$) |
| *Assumptions* | None | |
|  | **Survey** | **Breeding program** |
| *Trait* | Sweetpotato weevil resistance | Weevil damage |
| *Definition* | Percentage of plants infected with Weevil | Overall assessment of weevil damage based on inspection of the harvested roots (on a scale from 1 to 9) |
| *Unit* | 1% | 1 score |
| *Matching units* | 0.0% (no plants infected) | 1 (no damage) |
|  | 12.5% | 2 |
|  | 25.0% | 3 (minor damage) |
|  | 37.5% | 4 |
|  | 50.0% | 5 (moderate damage) |
|  | 62.5% | 6 |
|  | 75.0% | 7 (heavy damage) |
|  | 87.5% | 8 |
|  | 100.0% (all plants affected) | 9 (severe damage) |
| *Transforming breeding program* $\sigma_{a}$ *to survey unit* | 12.50%  3.28% | 1 score  0.26 score (= $\sigma_{a}$) |
| *Assumptions* | The correlation between the two units is assumed to be 1. | |
|  | **Survey** | **Breeding program** |
| *Trait* | Sweetpotato virus disease (SPVD) resistance | SPVD resistance |
| *Definition* | Percentage of infected plants | Resistance against SPVD |
| *Unit* | 1% | % |
| *Matching units* | 0% (no plants infected)  100% (all plants infected) | 100% (full resistance, no plants diseased  0% (no resistance, all plants diseased) |
| *Transforming breeding program* $\sigma_{a}$ *to survey unit* | 1.07% | 1.07% (= $\sigma_{a}$) |
| *Assumptions* | None | |
|  | **Survey** | **Breeding program** |
| *Trait* | Vine survival (under stressful conditions) | Plant establishment |
| *Definition* | Vine survival after planting under stressful conditions | Percentage of established plants (compared to planted plants) 4-6 weeks after planting |
| *Unit* | 1% | Percentage |
| *Matching units* | 100% (all vines survived after planting)  0% (no vines survived after planting) | 100% (all planted plants also established)  0% (none of the planted plants established) |
| *Transforming breeding program* $\sigma_{a}$ *to survey unit* | 9.66% | 9.66% (= $\sigma_{a}$) |
| *Assumptions* | Timing for survey and breeding program trait does not affect the parameters. | |
|  | **Survey** | **Breeding program** |
| *Trait* | Storage shelf life | Storage (data from Ghana) |
| *Definition* | Number of days after harvest before root spoilage | Number of weeks until general appearance drops below score 3 |
| *Unit* | 1 day | Number of weeks |
| *Matching units* | 7 days | 1 week |
| *Transforming breeding program* $\sigma_{a}$ *to survey unit* |  | (= $\sigma_{a}$) |
| *Assumptions* | Data not useful as general appearance score (1 - 9; 1 = not usable and 9 = field fresh or no symptoms of deterioration) was >7 at 13 weeks of storage (= end of evaluation). Obtained data comes from a different environment (Ghana). | |
|  | **Survey** | **Breeding program** |
| *Trait* | Root shape | Root appearance |
| *Definition* | Percentage of roots of desirable shape (score 5)  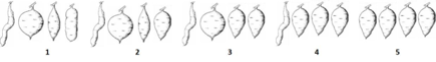 | Overall assessment of storage root form based on inspection of the harvested roots (on a 1 to 9 scale) |
| *Unit* | 1% | 1 score |
| *Matching units* | 100.0% (all roots have the desirable shape)  87.5%  75.0%  62.5%  50.0%  37.5%  25.0%  12.5%  0.0% (none of the roots have the desirable shape) | 1 (excellent)  2  3 (good)  4  5 (fair)  6  7 (poor)  8  9 (terrible) |
| *Transforming breeding program* $\sigma_{a}$ *to survey unit* | 12.50%  7.82% | 1 score  0.63 score (= $\sigma_{a}$) |
| *Assumptions* | Score 5 (in survey unit) is the desirable/excellent (in breeding program unit) root shape.  The correlation between the two units is assumed to be 1. | |
|  | **Survey** | **Breeding program** |
| *Trait* | Days to maturity | Maturity period (days) |
| *Definition* | Number of days to root maturity | Number of days to root maturity |
| *Unit* | 1 day | 1 day |
| *Matching units* | 1 day | 1 day |
| *Transforming breeding program* $\sigma_{a}$ *to survey unit* | 2.93 days | 2.93 days (= $\sigma_{a}$) |
| *Assumptions* | Genetic standard deviation of days to maturity (2.93) was calculated as 9.75 days (phenotypic standard deviation based on the provided means of maturity period (in days) of 22 varieties^a^) multiplied by its heritability (assumed to be 0.3).  ^a^Tororo 3, Bwanjule, New Kawogo, Tanzania, Wagabolige, Sowola, NASPOT 1, NASPOT 2, NASPOT 3, NASPOT 4 , NASPOT 5, NASPOT 6, Kakamega (SPK004), Ejumula, Dimbuka-Bukulula, NASPOT 7, NASPOT 8, NASPOT 9 O (Vita), NASPOT 10 O (Kabode), NASPOT 11, NASPOT 12 O, NASPOT 13 O | |
|  | **Survey** | **Breeding program** |
| *Trait* | Flesh sweetness | Sweetness (data from Ghana) |
| *Definition* | Sweetness of the flesh (linked to sugariness) on a scale from 1 to 5 (non-sweet to very sweet) | Sweetness scored by a sensory panel on a scale from 0 to 9 (non-sweet to very sweet) |
| *Unit* | 1 score | 1 score |
| *Matching units* | 5 (very sweet)  4.56  4.11  3.67  3.22  2.78  2.33  1.89  1.44  1 (non-sweet) | 9 (very sweet)  8  7  6  5  4  3  2  1  0 (non-sweet) |
| *Transforming breeding program* $\sigma_{a}$ *to survey unit* | 0.44 (= 4/9)  0.60 | 1 score  1.37 (= $\sigma_{a}$) |
| *Assumptions* | Obtained data comes from a different environment (Ghana). Assumed to be comparable to the Ugandan environments.  The correlation between the two units is assumed to be 1. | |
|  | **Survey** | **Breeding program** |
| *Trait* | Skin smoothness | No data available? |
| *Definition* | Smoothness of the skin on a scale from 1 to 5 (very rough to very smooth – see Matching units 1to 5 below)  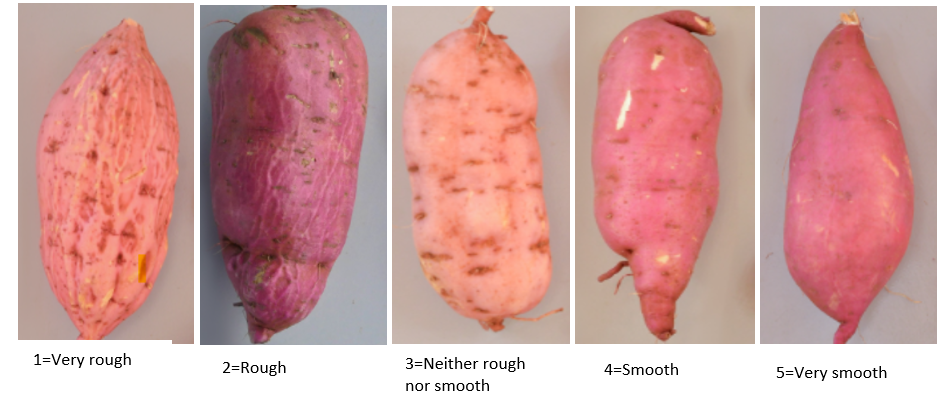 |  |
| *Unit* | 1 score |  |
| *Matching units* | 5 (very smooth)  4 (smooth)  3 (neither rough nor smooth)  2 (rough)  1 (very rough) |  |
| *Transforming breeding program* $\sigma_{a}$ *to survey unit* | … score | (= $\sigma_{a}$) |
| *Assumptions* |  | |
|  | **Survey** | **Breeding program** |
| *Trait* | Hardness of roots after cooking | Toughness/area under the curve (AUC; data from RTBfoods) |
| *Definition* | Root hardness after cooking on a scale from 1 to 5 (very moist to very dry) | Toughness/texture of the roots was scored on a linear scale from 1 to 9 |
| *Unit* | 1 score | 1 score |
| *Matching units* | 5 (very dry)  4.5  4.0  3.5  3.0  2.5  2.0  1.5  1 (very moist) | 9 (very hard)  8  7  6  5  4  3  2  1 (very soft) |
| *Transforming breeding program* $\sigma_{a}$ *to survey unit* | 0.50 (= 4/8)  0.61 | 1 score  1.21 (= $\sigma_{a}$) |
| *Assumptions* | (Poor to no correlation between texture and dry matter in certain varieties (R^2^ = 0.198) based on results from RTBfoods project). Using data on texture based on an objective analysis (AUC ranged from 1,200 (very soft) to 12,000 (very hard), with $\sigma_{G}^{2}$ = 2,649,679) rendered a similar emphasis on the trait as compared to toughness/texture score on a scale of 1 to 9 (data not shown).  The correlation between the two units is assumed to be 1. | |
|  | **Survey** | **Breeding program** |
| *Trait* | Flesh colour of roots | Flesh colour |
| *Definition* | Increase in colour intensity on a scale from 1 to 5 (low to high intensity); independent of colour) 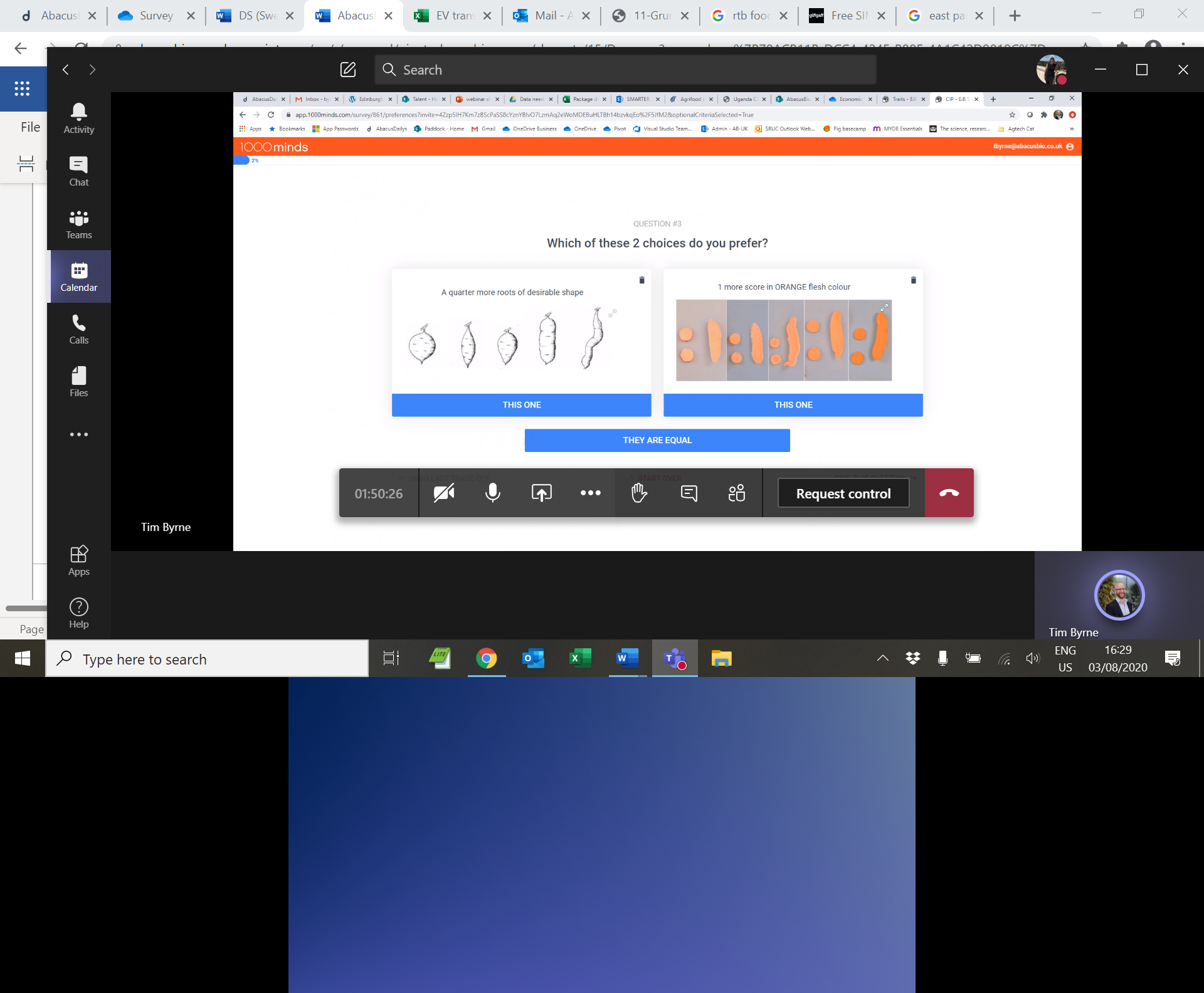 1 2 3 4 5 | Flesh colour of the roots on a scale from 1 to 30 (representing a linear score from white to orange) |
| *Unit* | 1 score | 1 score |
| *Matching units* | 5 (high intensity)  4  3 (medium intensity)  2  1 (low intensity) | 30 (orange)  1 (white) |
| *Transforming breeding program* $\sigma_{a}$ *to survey unit* | 0.14 score (= 4/29)  0.93 score | 1 score  6.76 score (= $\sigma_{a}$) |
| *Assumptions* | The correlation between the two units is assumed to be 1. | |
